# Delayed puberty – clinical approach and management

**DOI:** 10.1186/1687-9856-2015-S1-O20

**Published:** 2015-04-28

**Authors:** Pik To Cheung

**Affiliations:** 1 The Unviersity of Hong Kong, Hong Kong, China

## 

Delayed puberty, while not uncommonly a variant of normal pubertal physiology, could be an indicator of a wide range of less common but important clinical disorders. Congenital pathologies may involve defective developments of the gonads, pituitary gonadotrophs or hypothalamic GnRH neurons. Acquired pathologies could likewise be acting at each level. Clinical diagnostic evaluation to delineate a good range of these disorders and the respective managements will be covered in this session.

